# Supplementary material for: AffyMAPSDetector: a software tool to characterize Affymetrix GeneChip™ expression arrays with respect to SNPs
Source: BMC Bioinformatics. 2007 Jul 30;8:276. doi: 10.1186/1471-2105-8-276 (PMC1959249; doi:10.1186/1471-2105-8-276)
Supplement: Additional file 13 — AffyMAPSDetector v1 source code. AffyMAPSDetector source code. [file 1471-2105-8-276-S13.zip › AffyMAPSDetector_v1_src/doc/allclasses-frame.html]

All Classes


**All Classes**
  

AffyMapsDetectorFrame
  
AffyMapsDetectorLauncher
  
AffyMapsDetectorUI
  
AppIcons
  
ColorGradient
  
ComplementDnaSeq
  
Constant
  
CrossHbCheckFrame
  
DataState
  
DuplicateRemover
  
ErrorMessageBox
  
FileLoaderWizardDialog
  
GeneAnnotLoaderWizard
  
GeneInfo
  
GuiUtils
  
NullWizardPanel
  
OptimizedSnpDetector
  
PmMmHeatMapViewer
  
PmMmHeatmapViewerModel
  
PmMmHeatmapViewerModel.RowComparatorForDataSorting
  
PmMmMatrixLoader
  
Probe
  
ProbeSet
  
ResourceLoader
  
SnpDetector
  
SnpFinder
  
SnpInfo
  
SnpMatcher
  
StatusEvent
  
*StatusEventListener*
  
StepOnePanel
  
StepThreePanel
  
StepTwoPanel
  
Wizard
  
WizardAdapter
  
WizardContext
  
*WizardListener*
  
WizardPanel
  
WizardUtils
